# Deformation of the Outer Hair Cells and the Accumulation of Caveolin-2 in Connexin 26 Deficient Mice

**DOI:** 10.1371/journal.pone.0141258

**Published:** 2015-10-22

**Authors:** Takashi Anzai, Ichiro Fukunaga, Kaori Hatakeyama, Ayumi Fujimoto, Kazuma Kobayashi, Atena Nishikawa, Toru Aoki, Tetsuo Noda, Osamu Minowa, Katsuhisa Ikeda, Kazusaku Kamiya

**Affiliations:** 1 Department of Otorhinolaryngology, Juntendo University Faculty of Medicine, Tokyo 113–8421, Japan; 2 Department of Cell Biology, Japanese Foundation for Cancer Research, Cancer Institute, Tokyo 135–8550, Japan; 3 Team for Advanced Development and Evaluation of Human Disease Models, RIKEN BioResource Center, Tsukuba 305–0074, Japan; Universidade Federal do ABC, BRAZIL

## Abstract

**Background:**

Mutations in *GJB2*, which encodes connexin 26 (Cx26), a cochlear gap junction protein, represent a major cause of pre-lingual, non-syndromic deafness. The degeneration of the organ of Corti observed in Cx26 mutant—associated deafness is thought to be a secondary pathology of hearing loss. Here we focused on abnormal development of the organ of Corti followed by degeneration including outer hair cell (OHC) loss.

**Methods:**

We investigated the crucial factors involved in late-onset degeneration and loss of OHC by ultrastructural observation, immunohistochemistry and protein analysis in our Cx26-deficient mice (Cx26^f/f^P0Cre).

**Results:**

In ultrastructural observations of Cx26^f/f^P0Cre mice, OHCs changed shape irregularly, and several folds or notches were observed in the plasma membrane. Furthermore, the mutant OHCs had a flat surface compared with the characteristic wavy surface structure of OHCs of normal mice. Protein analysis revealed an increased protein level of caveolin-2 (CAV2) in Cx26^f/f^P0Cre mouse cochlea. In immunohistochemistry, a remarkable accumulation of CAV2 was observed in Cx26^f/f^P0Cre mice. In particular, this accumulation of CAV2 was mainly observed around OHCs, and furthermore this accumulation was observed around the shrunken site of OHCs with an abnormal hourglass-like shape.

**Conclusions:**

The deformation of OHCs and the accumulation of CAV2 in the organ of Corti may play a crucial role in the progression of, or secondary OHC loss in, *GJB2*-associated deafness. Investigation of these molecular pathways, including those involving CAV2, may contribute to the elucidation of a new pathogenic mechanism of *GJB2*-associated deafness and identify effective targets for new therapies.

## Introduction

Hereditary deafness is one of the most common congenital diseases. [1, 2]. Approximately one in 1000 children is affected by severe hearing loss at birth or during early childhood, which is defined as pre-lingual deafness [3, 4], with approximately half of these cases attributable to genetic causes [5]. Among the >100 known forms of non-syndromic deafness with identified genetic loci, by far the most common and best characterized is the one associated with *GJB2* (OMIM 121011), the gene encoding connexin 26 (Cx26) [6]. We previously reported the generation of mouse models for Cx26-associated deafness and their molecular pathophysiologies. We recently showed that the delayed programmed cell death observed in Cx26 mutant mice resulted in an abnormal shapes for the organ of Corti [7], and mutation of Cx26 resulted in a drastic disruption and reduction in the gap junction plaque as well as an ion transport disorder. We also found that the corresponding upregulation and isoform shift of caveolin (CAV) may underlie these disruptions [8]. It has been reported that histopathologic evaluation of the human temporal bone in Cx26-related hearing loss revealed near-total degeneration of hair cells in the organ of Corti [9]. Both a Cx26 dominant-negative model and a conditional knockout model developed secondary degeneration [10–13], which could be rescued by gene transfer with wild-type *Gjb2* [14]. The mechanism underlying secondary outer hair cell (OHC) degeneration remains unknown. Because the mammalian inner ear largely lacks the capacity to regenerate OHCs [15], the mutant Cx26–associated degeneration leads to irreversible hearing loss. Even if certain therapies, drugs, or a superior cochlear implant is developed for Cx26-associated deafness, these irreversible changes may counter any attempt to treat the hearing loss. Here we demonstrate the deformation of OHCs in mice with Cx26-associated hearing loss and investigate the factors that contribute to the secondary degeneration of OHCs.

## Materials and Methods

### Animals and ethics statement

The care, maintenance, and treatment of animals in these studies followed protocols approved by the Institutional Animal Care and Use Committee at Juntendo University (Permit Number: 270201).

As we previously reported [8], otic vesicle—specific Cx26 knockout mice were generated by breeding Cx26^f/f^ mice with mice that expressed the Cre recombinase gene under the control of the P0 gene promoter (P0Cre mice on the C57BL/6J background). Cx26^f/f^ on a C57Bl/6J background in littermates was always used as the control for the Cx26^f/f^P0Cre mice. Mouse genotypes were verified via polymerase chain reaction. To the extent possible, we minimized the number of animals used and their suffering.

### Transmission electron microscopy

Animals were deeply anesthetize and perfused intracardially with phosphate-buffered saline, followed by 2% paraformaldehyde and 2% glutaraldehyde in cacodylate buffer. The cochleae were resected and flushed with the fixative for 2 h at room temperature. After washing, the specimens were post-fixed for 1.5 h with 2% osmium tetroxide in phosphate buffer and then were dehydrated through a graded ethanol series and embedded in Epon. Horizontal sections of the surface of the cochlear membrane labyrinth were made, stained with uranyl acetate and lead citrate, and examined by electron microscopy (Model H-7700, Hitachi).

### Immunohistochemistry

Mice were anesthetized and killed, and inner-ear tissues were then removed. The cochleae were further dissected and fixed in 4% paraformaldehyde. Immunofluorescence staining with antibodies against CAV2 (mouse IgG; BD) and Prestin (goat IgG; Santa Cruz) along with DAPI (Vector Laboratories) was performed on whole-mount preparations of the carefully resected organ of Corti or cochlear cryosections (7 μm). We incubated the tissues in the antibody solutions for 1 h at room temperature after blocking with 2% bovine serum albumin in phosphate-buffered saline. Fluorescence confocal images were obtained with a LSM510-META confocal microscope (Carl Zeiss). CAV2 was labeled with Alexa 488 (mouse IgG; Life Technologies) and observed by confocal laser microscopy using a 488-nm laser. Prestin was labeled with Alexa 633 (mouse IgG; Life Technologies) and observed by confocal microscopy using a 633-nm laser. Some of the red signals in the figures reflect the pseudo-color of the Alexa 488 signal.

### Western blotting

Mouse cochlear proteins were extracted with T-PER^®^ Tissue Protein Extraction Reagent (Thermo Scientific) from at least six cochleae that included the organ of Corti, lateral wall, and stria vascularis. The proteins were resolved by SDS-PAGE using mini-PROTEAN TGX gradient gels (4–20% polyacrylamide; Bio-Rad Laboratories, Inc.) and then transferred to a polyvinylidene difluoride membrane (Amersham Hybond-P; GE Healthcare). After blocking, each membrane was processed through sequential incubations with anti-CAV2 (1:500, Sigma Aldrich) and monoclonal anti-β-actin (1:1500; Sigma Aldrich) with horseradish peroxidase—conjugated anti-rabbit or anti-mouse IgG (1:40,000; GE Healthcare) as the secondary antibody. Amersham ELC Prime Western Blotting Detection Reagent (GE Healthcare) was then used for visualization, and the signal was observed by Image Quant LAS 4000 (Fujifilm). Each experiment was carried at least three times. Densitometric analysis of band intensities was performed with Multi Gauge Ver3.2. The data were normalized to the corresponding β-actin levels and expressed relative to the amount present in each littermate control and were compared using the Student’s *t*-test (Excel).

### Image reconstruction

Coronal images of OHCs were constructed using *z*-stacked confocal images with IMARIS software (Bitplane).

### Statistics

A one-tailed Student’s *t*-test, with a significance criterion of P < 0.05, was used to compare numbers of cells or the level of CAV2 among samples.

## Results

In this study, we performed ultrastructural and protein analysis of cochlea tissue using Cx26^f/f^P0Cre mice to investigate the mechanism and factors contributing to secondary degeneration of OHCs.

**Fig 1:**
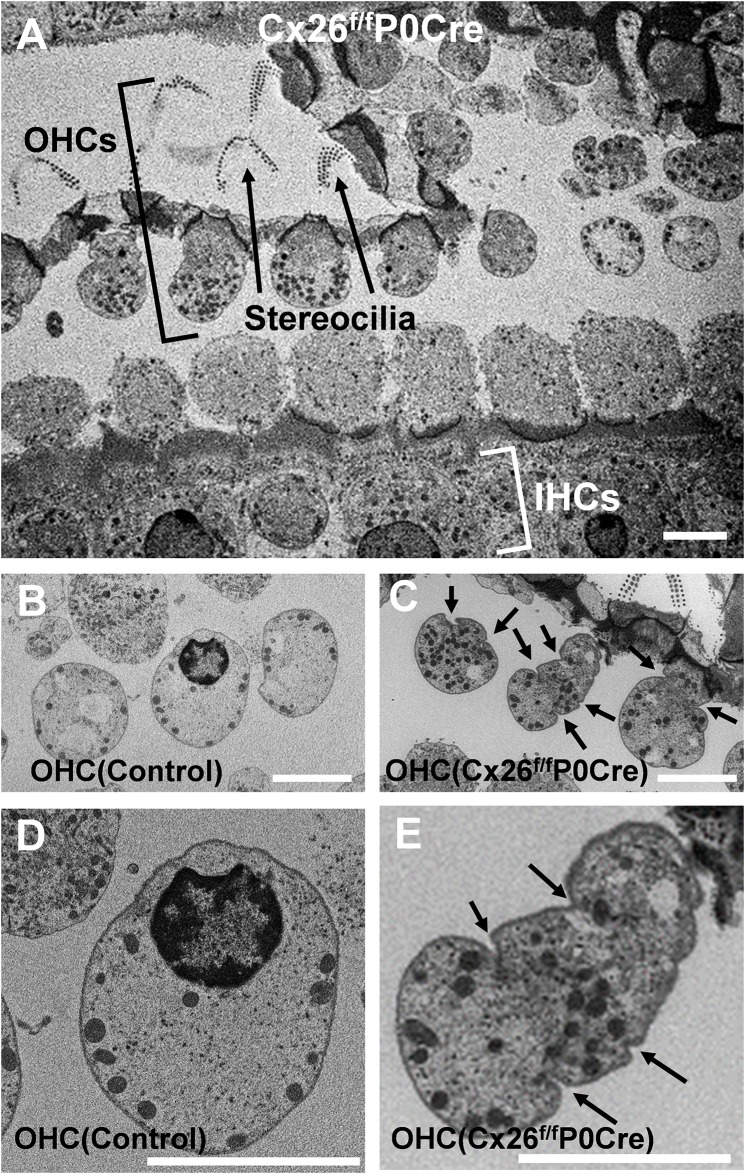
Transmission electron micrographs of a horizontal section of OHCs. Micrographs around apical part of cochlea of 5-week-old control (B, D) and Cx26^f/f^P0-Cre (A, C, E) mice. Although, round-shaped OHCs were observed in control mice (B, D), deformed OHCs were observed in Cx26^f/f^P0Cre mice (A, C, E). OHCs in Cx26^f/f^P0-Cre mice exhibited altered shapes, and several irregular folds or notches (arrows) were observed in the plasma membrane (C-E). Scale bars, 10 μm. IHCs, inner hair cells; OHCs, outer hair cells.

Horizontal ultrathin sections of the organ of Corti of 5-week-old Cx26^f/f^P0Cre with the control littermate are shown in Fig 1A. Although normal/round-shaped OHCs were observed in control mice (Fig 1B and 1D), OHC remarkable deformation was observed in Cx26^f/f^P0Cre mice (Fig 1C and 1E). The OHCs of control mice had a smooth plasma membrane (Fig 1B and 1D), whereas OHCs of Cx26^f/f^P0Cre mice had altered/irregular shapes, and several notches or folds were observed in the plasma membrane (arrows in Fig 1C and 1E). These deformations were detected in horizontal sections of OHCs, although they were never detected in our conventional mid-modiolar sections [16, 17].

**Fig 2:**
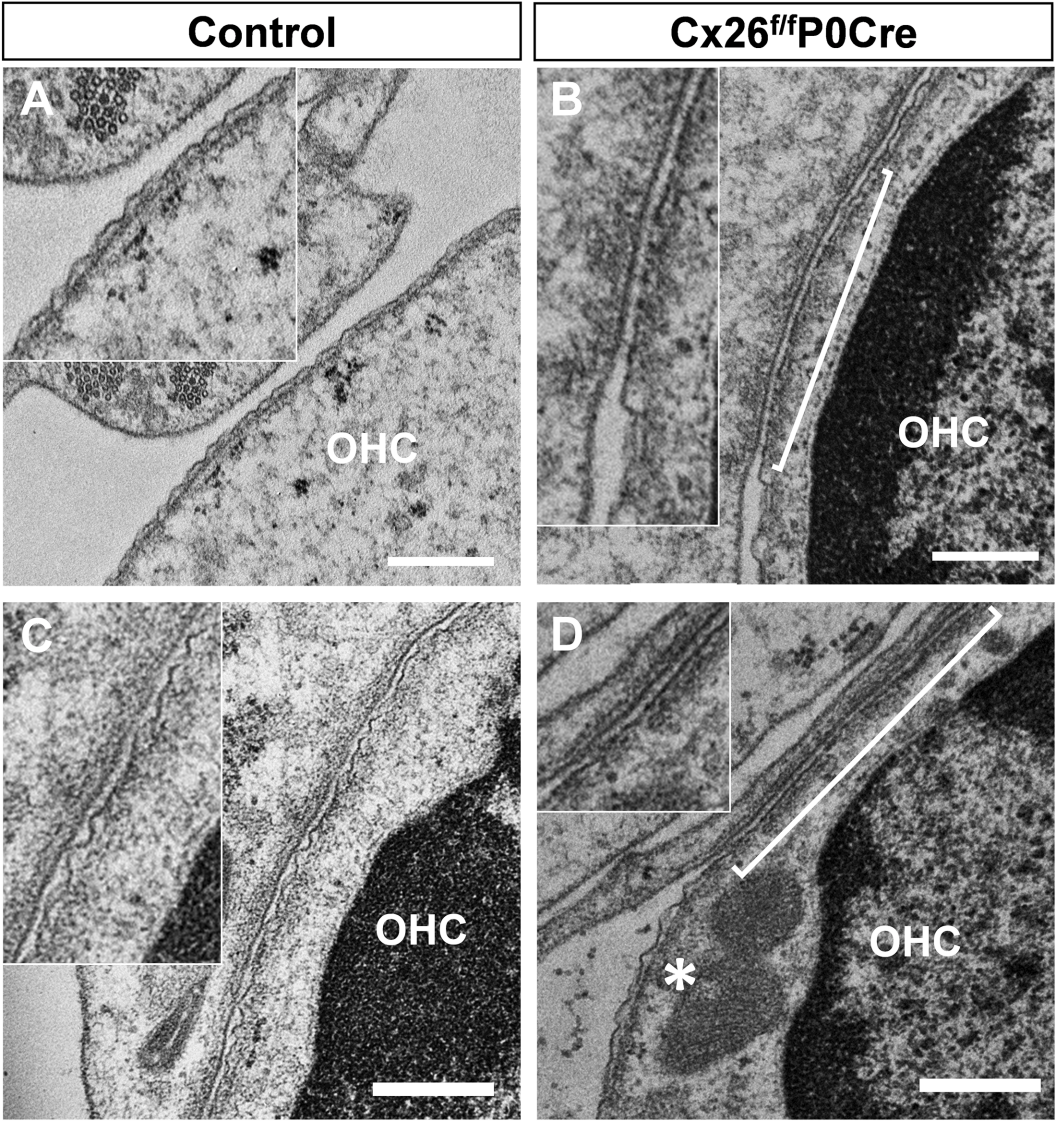
Ultrastructure of the plasma membrane of OHCs. Transmission electron micrographs of OHCs. Ultrastructure of the plasma membrane of OHCs around apical part of cochlea in 5-week-old Cx26^f/f^P0Cre mice (B and D) and littermate controls (A and C). Horizontal ultrathin sections of the organ of Corti, including transverse section of OHCs, show the wavy surface of the plasma membrane that is thought to indicate the structure of the cortical lattice (A and C). In Cx26^f/f^P0Cre mice, a wavy surface structure was less apparent, and flat surfaces (brackets in B and D) were observed among the normal wavy surface regions of the plasma membrane (asterisk in D) at several points in OHCs. Scale bars, 500 nm. OHCs, outer hair cells.

A wavy cell surface, which is thought to indicate a normal cortical lattice, was observed by transmission electron microscopy of horizontal ultrathin sections focused on the ultrastructure of the plasma membrane of OHCs. In Cx26-deficient mice, however, the wavy surface structure of the OHC membrane was not always apparent and indeed a flat surface (bracket in Fig 2D) was observed at several places along the mutant OHC plasma membrane. As was the case for the above-mentioned OHC deformations, a mixed flat/wavy surface ultrastructure was detected only in horizontal sections of OHCs, and this was never detected in our conventional mid-modiolar sections [16, 17].

**Fig 3:**
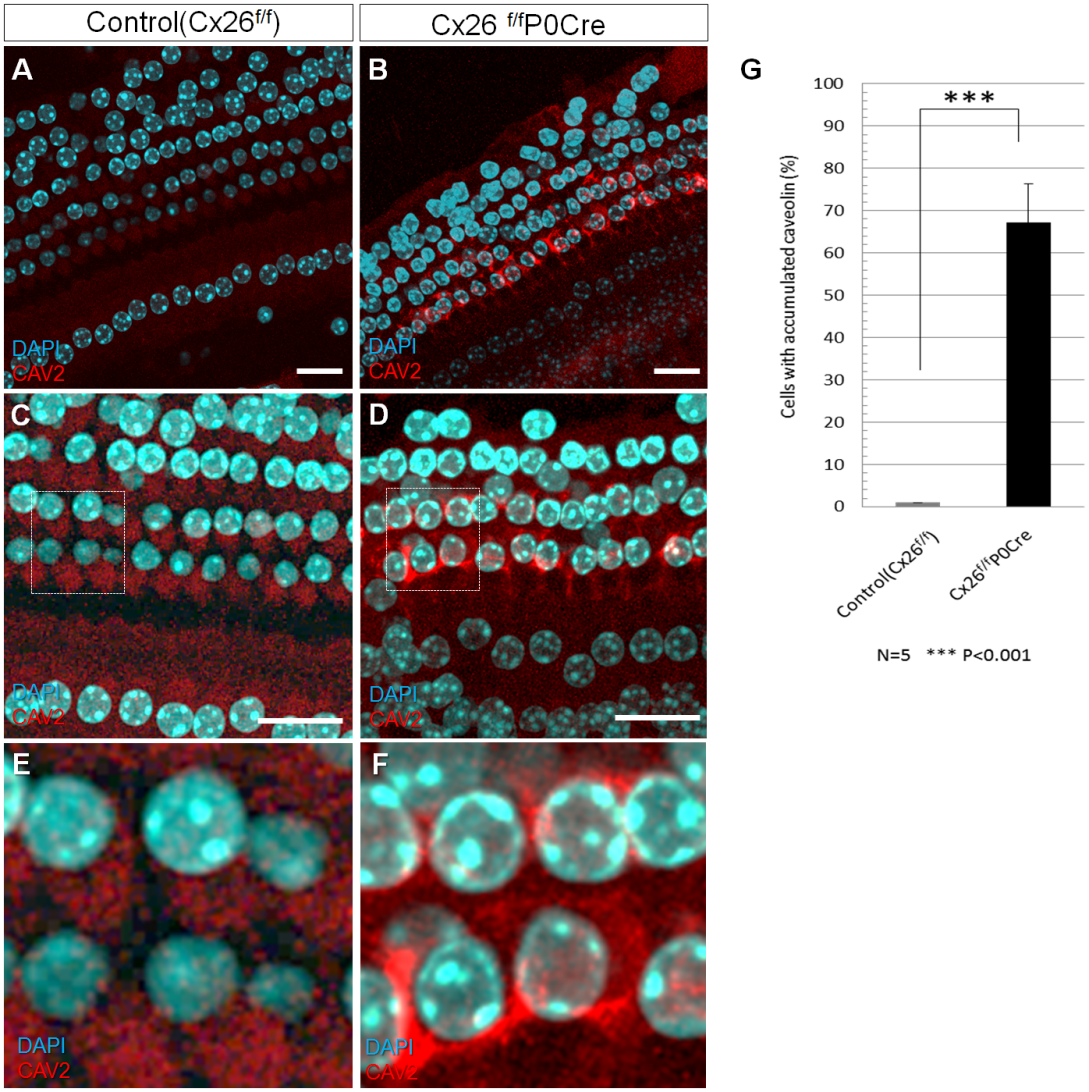
CAV2 accumulation in the organ of Corti. (A-F) Immunofluorescence staining for CAV2 in the organ of Corti around apical part of cochlea in 3-week-old Cx26^f/f^P0Cre mice with *GJB2*-associated deafness and in littermate controls. Whole-mount cochleae were fixed and immunolabeled with anti-CAV2 (red). Nuclei were counterstained with DAPI (blue). In contrast to the controls, notable accumulation of CAV2 was observed at the organ of Corti in Cx26^f/f^P0Cre cochlea**e**. (G) shows the mean percentage of cells with accumulated CAV2 in control and Cx26^f/f^P0Cre cochleae. There was a statistically significant difference between the control and Cx26^f/f^P0Cre mice. Values represent the mean ± SE (n = 5 mice). ***P = 3.72 × 10^−5^, Student’s *t*-test.

We investigated the factors contributing to the observed deformation of OHCs and the secondary degeneration at the organ of Corti of Cx26^f/f^P0Cre mice. After several protein analysis of mutant cochlea from these mice, we found increased expression and abnormal localization of CAV2 in the organ of Corti. Although, only diffuse labeling of CAV2 was observed in the organ of Corti of control mice (Fig 3A, 3C and 3E), accumulation of CAV2 was apparent in Cx26^f/f^P0Cre mice (Fig 3B, 3D and 3E). In particular, this accumulation was notable in OHCs, Deiter’s cells, and pillar cells. Moreover, the number of cells exhibiting abnormal CAV2 accumulation was significantly greater in Cx26^f/f^P0Cre mice compared with control mice (Fig 3G).

**Fig 4:**
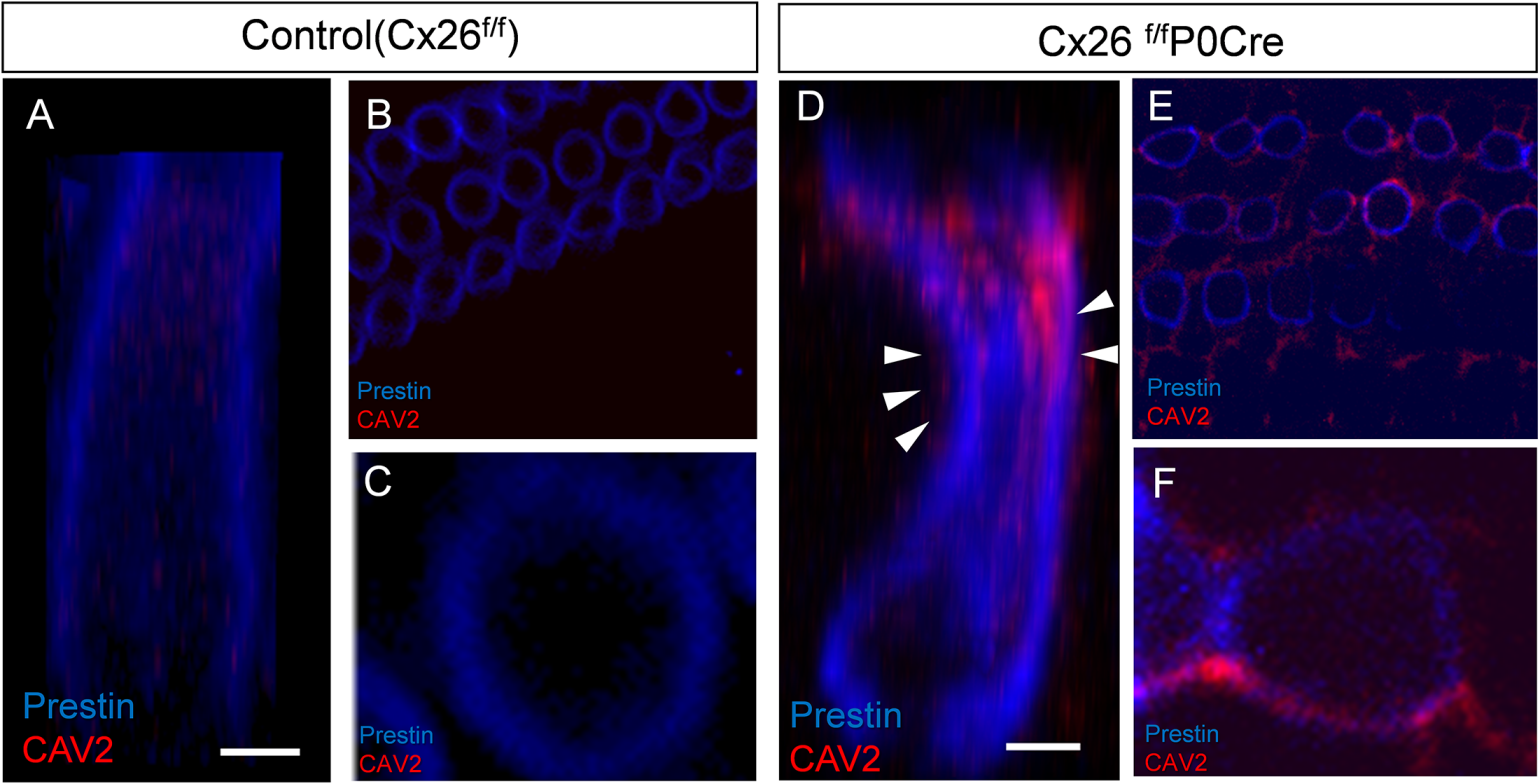
Subcellular localization of CAV2 in OHCs. Accumulation of CAV2 was observed in OHCs around apical part of cochlea and colocalized with prestin in 3-week-old control and Cx26^f/f^P0Cre mice. *z*-stacks of images were collected at 0.5-μm intervals (B, C, E, F), and the images of OHCs were reconstructed with graphics software (A, D). Double labeling for CAV2 and prestin revealed that OHCs of Cx26^f/f^P0Cre mice had an altered, hourglass-like shape and that CAV2 accumulated near the basolateral plasma membranes. In contrast, CAV2 accumulation was mainly observed surrounding the shrunken site of OHCs in Cx26^f/f^P0Cre mice (arrowheads in D). OHCs, outer hair cells.

To detect the lateral plasma membrane of OHCs, we utilized the OHC-specific protein prestin. In the reconstructed image of the mid-modiolar section, OHCs of Cx26^f/f^P0Cre mice had an altered, hourglass-like structure (Fig 4A and 4D), and CAV2 accumulated around the basolateral membranes (Fig 4D, 4E and 4F). In control OHCs, CAV2 localized diffusely in the cytoplasm (Fig 4A). In Cx26^f/f^P0Cre OHCs, however, accumulation of CAV2 was mainly observed around the shrunken site of OHCs (arrowheads in Fig 4D).

**Fig 5:**
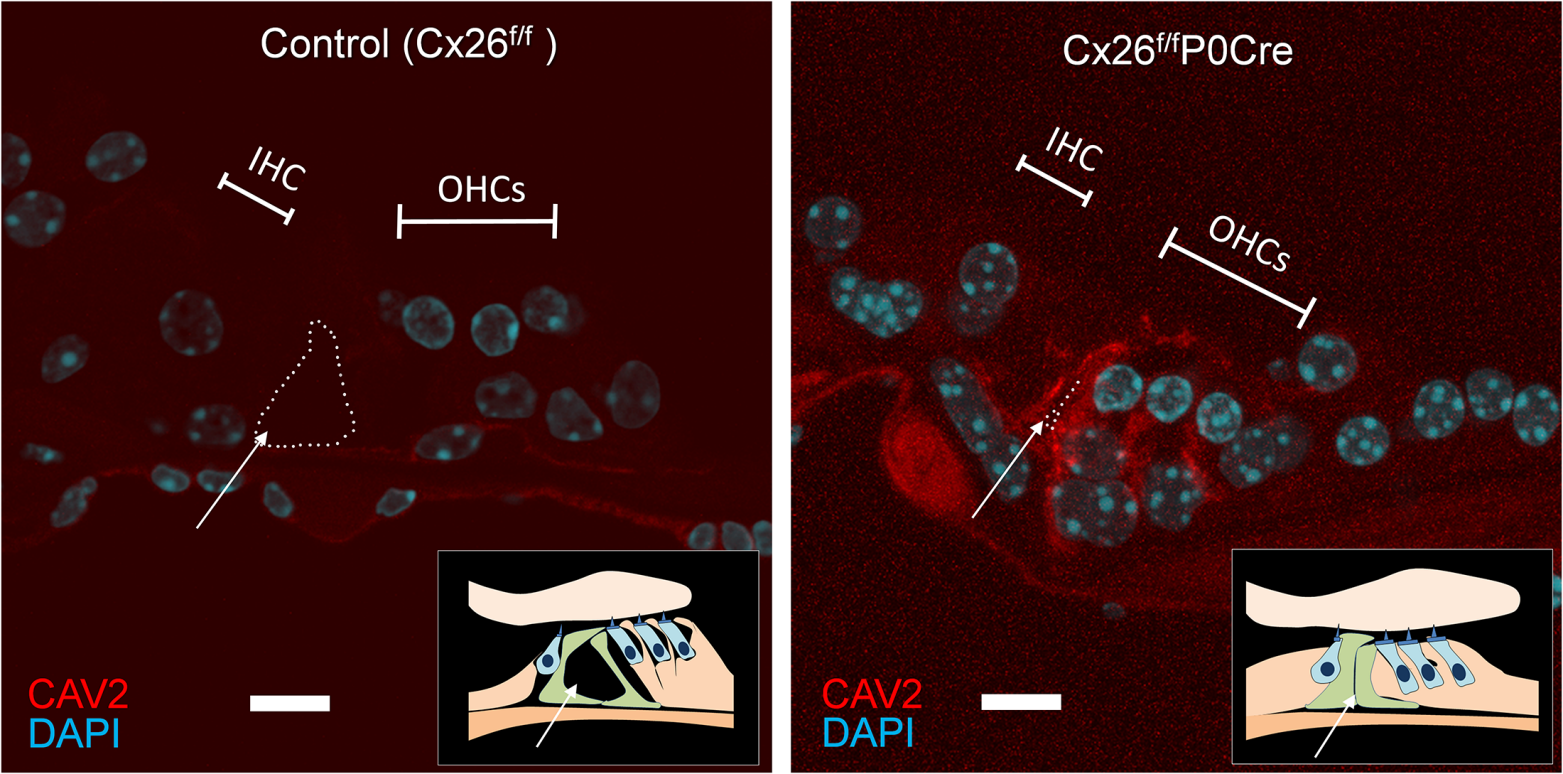
Immunolabeling of CAV2 in cryosections of the organ of Corti in 3-week-old control and Cx26^f/f^P0-Cre mice. Cryosections of the organ of Corti around apical part of cochlea were immunolabeled with anti-CAV2 (red). Nuclei were counterstained with DAPI (blue). In contrast to the controls, the TC (dotted line with an arrow) was closed, and notable accumulation of CAVs was observed in OHCs and supporting cells. In particular, such accumulation was observed in cells surrounding the closed TC in Cx26^f/f^P0Cre cochleae. Scare bars, 10 μm. IHC, inner hair cell; OHCs, outer hair cells; TC, tunnel of Corti.

**Fig 6:**
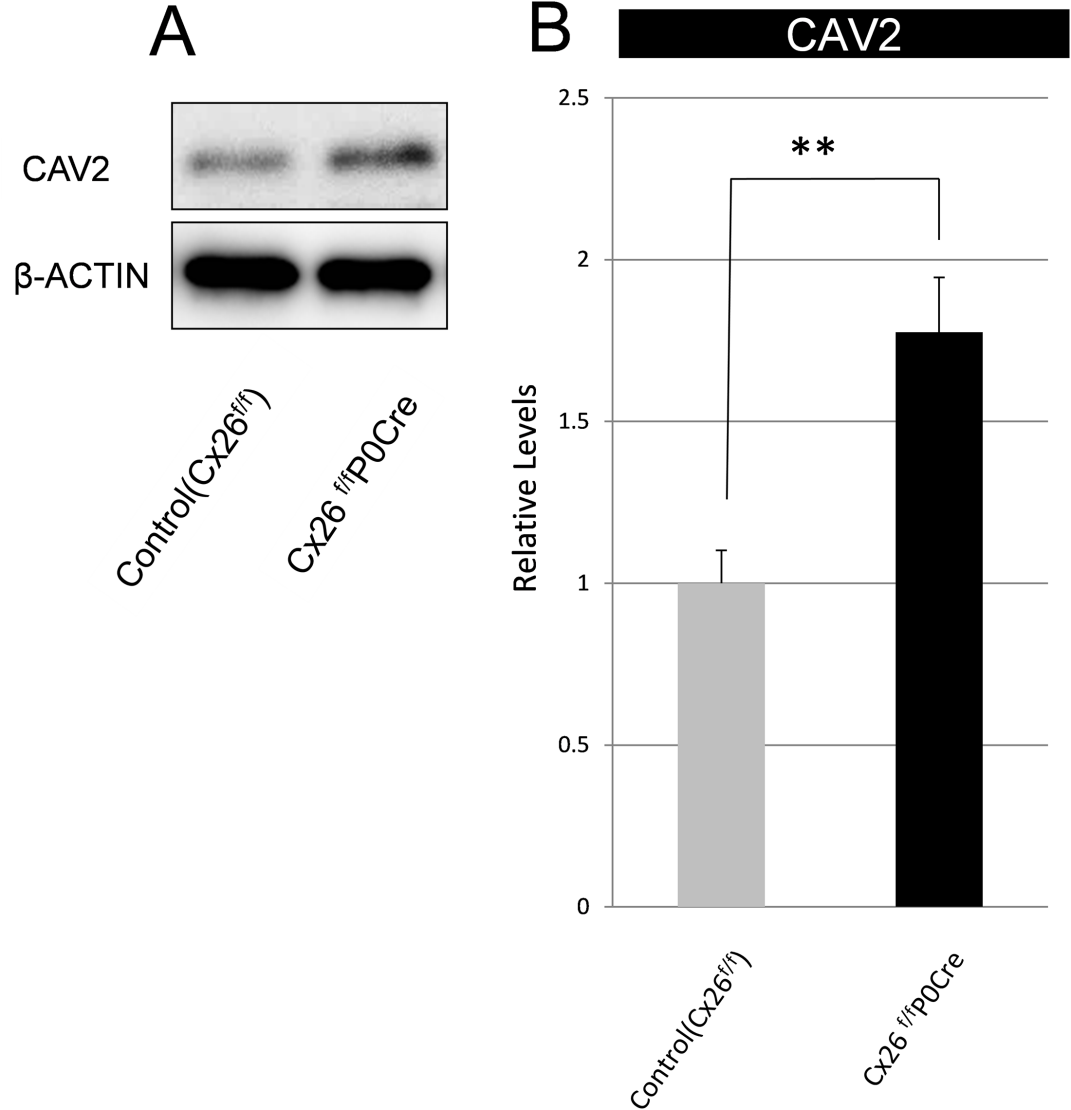
Protein expression level of CAV2. (A) Western blotting revealed an increase in CAV2 level in Cx26^f/f^P0Cre mice at postnatal day 21. (B) CAV level was normalized to that of β-actin and is expressed as relative to the amount present in each littermate control. Values represent the mean ± SE (n = 6 for control, n = 5 for Cx26^f/f^P0Cre). **P = 2.5×10^−3^ for CAV2, Student’s *t*-test.

Fig 5 shows the immunolabeling for CAV2 in cryosections of the organ of Corti in 3-week-old control and Cx26^f/f^P0Cre mice. Although an open tunnel of Corti (TC) was observed in control mice (dotted line and arrow in Fig 5A), a closed TC was observed in Cx26^f/f^P0Cre mice (dotted line and arrow in Fig 5B). CAV2 accumulation, as shown in Figs 3 and 4, was also observed in cryosections of the organ of Corti. In particular, CAV2 accumulation was observed in cells surrounding the closed TC. (Fig 5B). In addition to our confocal analysis of CAV2 localization in the organ of Corti, western blotting revealed that the CAV2 level was significantly greater in Cx26^f/f^P0Cre mice (1.8-fold) compared with control mice (Fig 6).

## Discussion

Our results demonstrate the deformation of OHCs in Cx26-deficient mice using unconventional horizontal sections. It is thought that the overall cyto-architecture of the organ of Corti is essential for normal hearing. In our previous work, we showed that developmentally essential apoptosis in the organ of Corti was delayed in Cx26 mutant mice [7]. Moreover, several studies have demonstrated that mutation of Cx26 arrests TC development [16–19], which is thought to be associated with hearing loss. These studies show that disruption of the cyto-architecture of the organ of Corti may cause the deformation of OHCs. Moreover, the characteristic wavy surface structure of the plasma membrane of normal OHCs, which was thought to indicate cortical lattice (Fig 2), was not observed partially in OHCs of Cx26^f/f^P0Cre mice. We speculate that alteration of the structure of the cortical lattice may underlie the observed deformation of OHCs in our Cx26^f/f^P0Cre mice (Fig 1). We previously reported that OHCs are compressed and squeezed by the surrounding supporting cells in Cx26 mutant mice [17]. These mechanical forces may reduce the wavy structure and result in a flat plasma membrane. The cortical lattice may regulate OHC stiffness and/or electromotilty [20–24]. We reported that distortion-product otoacoustic emission could not be detected throughout development of Cx26 dominant-negative model mice [17]. It is thought that mechanical stress and abnormal cyto-architecture suppress the distortion-product otoacoustic emission response and cause substantial damage to OHCs [17]. This may ultimately lead to the degeneration of secondary OHCs in Cx26^f/f^P0Cre mice.

CAVs are integral plasma-membrane proteins and the principal structural components of the localized caveolae membrane and related to endocytosis, cholesterol transport, and various signal transduction processes [25]. Recent experiments have shown that overexpression or abnormal localization of CAVs delays wound healing or accelerates cellar aging in several organs (e.g., skin [26, 27], lung [28], heart [29], and eye [30]). Among the three members of the caveolin family (CAV1, CAV2 and CAV3), CAV1 and CAV2 are expressed in most cell types. CAV3 is only expressed muscle cells [31, 32]. A recent study revealed that CAV2 is the key protein that regulates cell proliferation [33]. The CAV family is thought to be one of the stress-induced protein families, and CAVs negatively regulate cell proliferation and cell cycle progression [34]. It was also reported that CAV1 and CAV2 levels are elevated in endothelial cells in a mouse model of traumatic brain injury [35]. Furthermore, shear stress causes translocation of CAV1 from caveolae to noncaveolae sites and induces ERK activation [29]. In our current study, notable accumulation of CAV2 was observed in OHCs and supporting cells in Cx26^f/f^P0Cre mice. In particular, this accumulation was observed in cells near the closed TC (Fig 5B) and the shrunken site of OHCs (Fig 4D). These facts indicate OHCs and supporting cell were received some mechanical stress and the OHC secondary degeneration might be associated with CAV2. These facts may suggest that, as a consequence of CAV2 accumulation, the OHCs experienced secondary degeneration.

This is the first report demonstrating the characteristic deformation of OHCs and the identification of certain factors that contribute to OHC degeneration in the organ of Corti of Cx26^f/f^P0Cre OR Cx26 mutant mice. Our study also suggests that CAVs in the organ of Corti may play a crucial role in the progression or secondary pathogenesis of *GJB2*-associated deafness. It has been reported that CAVs polymorphisms are associated with the risk of Meniere’s disease, which is a disease of the inner ear that manifests as episodic vertigo [36]. CAVs may be important for inner-ear homeostasis. Investigation of these molecular pathways, including those involving CAV2, may contribute to our understanding of the pathogenesis of *GJB2*-associated deafness and provide new information on effective targets for new therapies.
